# Characteristics of patients with schizophrenia switching from oral antipsychotics to once-monthly paliperidone palmitate (PP1M): a systematic review

**DOI:** 10.1186/s12888-024-05508-6

**Published:** 2024-01-19

**Authors:** Qian Li, Xin Li, Chong Ye, Miaomiao Jia, Tianmei Si

**Affiliations:** 1 Peking University Sixth Hospital, Peking University Institute of Mental Health, NHC Key Laboratory of Mental Health (Peking University), National Clinical Research Centre for Mental Disorders (Peking University Sixth Hospital), Beijing, China; 2 Xi’an Janssen Pharmaceutical Ltd, Chaoyang District, Beijing, China

**Keywords:** Schizophrenia, Antipsychotic agents, Paliperidone palmitate, Systematic review

## Abstract

**Background:**

The utilization of once-monthly paliperidone palmitate (PP1M) in schizophrenia treatment has increased due to its enhanced adherence and convenience. However, there is limited evidence on patient characteristics that may influence treatment outcomes when switching from oral antipsychotics (OAPs) to PP1M therapy. This systematic review aims to identify such patient characteristics and explore potential beneficial factors to aid healthcare professionals in clinical practice.

**Methods:**

A systematic literature search was conducted in the PubMed, Embase, and Cochrane Library databases up to July 19, 2022. Studies related to patients with schizophrenia who had been previously treated with OAPs and switched to PP1M were identified and included. Outcomes included the Positive and Negative Syndrome Scale (PANSS) total score, the clinical Global Impressions – Severity (CGI-S) score, the Personal and Social Performance (PSP) total score, and hospitalisation rate. Data were independently extracted and analysed. The results were presented through a narrative synthesis.

**Results:**

Eleven studies with a total of 4150 patients were included, identifying nine potential characteristics. The most commonly reported characteristics was patient’s prior treatment with OAPs, followed by the stage of disease, duration of illness (DI), ethnicity, reason for switching to PP1M, history of hospitalisation, time of start injection of PP1M, the PANSS and PSP total score at baseline. Patients in the acute stage, with a shorter DI, a less than 1-week time interval to PP1M injection, and a lower PANSS total score at baseline may have a trend on providing better improvements on PANSS total score. Acute stage and shorter DI also showed potential trends in reducing CGI-S score. Early initiation of PP1M, switching for reasons other than lack of efficacy, and a higher PSP score at baseline exhibited potential trends towards better PSP total score improvements.

**Conclusion:**

Our findings may suggest that patients in acute stage, with a shorter duration of illness, with early initiation of PP1M injection, and lower PANSS or PSP scores may trend towards better clinical results when transitioning to PP1M from OAPs. Further research is necessary to validate these potential associations and identify any unexplored characteristics. Such investigations are crucial for providing comprehensive clinical recommendations and informing treatment strategies in this context.

**Supplementary Information:**

The online version contains supplementary material available at 10.1186/s12888-024-05508-6.

## Introduction

Schizophrenia can detrimentally affect individuals' capacity to learn, function in employment, maintain self-care, and establish interpersonal connections [1, 2]. The social and economic consequences of schizophrenia are substantial, impacting patients, their families, and society as a whole [2]. Antipsychotic medications play a vital role in the treatment of schizophrenia, and generally, individuals with this condition necessitate ongoing, lifelong antipsychotic treatment to prevent symptom recurrence [3].

Ensuring compliance with antipsychotic medication is crucial for individuals with schizophrenia. Poor adherence to these medications elevates the risk of symptom relapses, hospitalizations, emergency room visits, and greater healthcare expenses [4, 5]. Monotherapy and utilizing the minimum effective dosage of antipsychotics are recommended treatment approaches for schizophrenia patients [6]. Typically, oral antipsychotics (OAPs) are commonly used [7, 8], yet achieving adherence remains challenging. Despite the influence of specific drugs, patient ethnicity, patient age, and the diverse criteria for defining satisfactory adherence, the reported non-adherence rate ranged from 21.7% to 70.2% across both inpatient and outpatient settings [9, 10].

Long-acting injectable antipsychotics (LAIs) have demonstrated advantages in enhancing treatment adherence compared to OAPs, potentially leading to improvements in symptoms and reducing the risk of symptom relapse and rehospitalization. These benefits are observed across various stages of schizophrenia, including first-episode cases, inadequate response to oral antipsychotics (OAPs), or a history of relapses [11]. LAIs are currently recommended for maintenance treatment or relapse prevention therapy to maintain a longer stable state. Once-monthly paliperidone palmitate (PP1M) is a long-acting, injectable aqueous suspension formulation of paliperidone, with a unique pharmacokinetic profile that enables both a rapid achievement of therapeutic plasma levels and a steady release for dose administration interval [12, 13]. The specific pharmacokinetic profile of paliperidone allows for the initiation of PP1M in patients experiencing acute symptoms of schizophrenia without the need for oral supplementation [12, 14].

Despite the advantages of LAIs, including PP1M, prevalence of their prescription is low compared to OAPs [15]. Globally, the utilisation rate of LAIs in Western countries is around 20-30% [16, 17]. In contrast, Asian countries exhibit a lower rate of approximately 18%, with China specifically demonstrating a usage rate of less than 1% [18]. The low utilization rate of LAIs may be influenced by multiple factors, including clinicians' awareness and patients' willingness to accept this treatment option [19–21]. These factors, in turn, may be influenced by various aspects, such as policies and population characteristics [21, 22]. Previous studies in China found that eliminating the need of daily medication, a shorter course of disease, a younger age, and more hospitalisations may be associated with willingness to accept LAIs [19, 23]. Whereas, high cost, fear of injection and lack of understanding may be associated with unwillingness [19]. From clinician perspectives, limited knowledge about and experience with LAIs, pragmatic barriers to use LAIs such as cost, storage, and staffing, a tendency to consider LAIs as a last-resort option for patients with a history of non-adherence, and beliefs about negative perceptions of patients regarding LAIs may shape their view on LAIs [21].

Given the recommendation of paliperidone palmitate as a treatment option for patients transitioning from OAPs, it would be beneficial to acknowledge the population characteristics that can influence its clinical utilization. A prior study investigated factors linked to improved clinical outcomes in patients transitioning to PP1M [24]. The results indicated that baseline PANSS and PSP scores, as well as their changes at week 5, may be associated with symptom reduction or functional improvements. However, the inclusion of Chinese patients in the study limited the generalizability of the results to a global scale. A systematic search of databases revealed a scarcity of evidence, particularly prognostic studies, focusing on influencing factors in this area. Therefore, our systematic review aims to identify and summarise the characteristics of patients with schizophrenia switching from OAPs to PP1M. We seek to provide valuable insights and references for clinical practice, shedding light on the specific patient features that may affect the treatment outcomes from the transition to PP1M.

## Methods

This systematic review was conducted following the guidelines outlined in the Preferred Reporting Items for Systematic Review and Meta-Analyses (PRISMA) [25].

### Search strategy

A systematic literature search was conducted in the PubMed, Embase, and Cochrane Library from database inception up to July 19, 2022. The search strategies utilized the combination of free-text terms and medical subject headings (MeSH), with the following keywords: ("Schizophrenia"[Mesh] OR Schizophreni*[tw]) AND (xeplion[tw] OR PP1M[tw] OR Sustenna[tw]) OR (("long-acting inject*"[tw] OR LAI[tw] OR"1 month*"[tw] OR inject*[tw]) AND ("Paliperidone Palmitate"[Mesh] OR paliperidone[tw] OR "9 Hydroxyrisperidone"[tw])). Website and citation searching were also employed.

### Eligibility criteria

Studies were included if they met the following criteria:

Population: patients diagnosed with schizophrenia, as defined by the original study, who had a history of receiving OAPs. There were no restrictions on patient’s age.

Intervention: patients with a prior history of OAP use and currently switching to PP1M.

Comparator: there were no limitations on comparators.

Outcomes: outcomes included the Positive and Negative Syndrome Scale (PANSS) total score, the Clinical Global Impressions – Severity (CGI-S) score, the Personal and Social Performance (PSP) total score, and hospitalisation rate.

Study design: randomised controlled trials (RCTs), controlled clinical trials (CCTs), single-arm trials, and observational studies.

To minimize the impact of LAIs, patients with a history of previous treatment with an LAI reported at baseline were excluded. In addition, studies were excluded if patients were treated with OAPs alone or with three/six-monthly paliperidone palmitate. Case reports, reviews and abstracts were excluded. Only studies reporting findings in English were included.

### Literature screening, data extraction and synthesis

Two reviewers screened articles based on article titles and abstracts. Potentially relevant articles were requested and inspected in detail using the full-text version where available. Disagreements were resolved by discussion, with assistance from a third reviewer if necessary. A PRISMA flow chart was constructed to illustrate the entire study-selection process. Data extraction was performed independently by two reviewers using a pre-defined, standardized form. Any disagreements were resolved by a third reviewer. The extracted data included study characteristics such as author, publication date, study design, sample size, and study outcomes. Due to the heterogeneity of the primary studies and the initial purpose of this systematic review, the outcomes were described narratively. We conducted a descriptive analysis of the data included in the study, dividing it into four sections based on different outcome indicators. Each section reported on the explored patient characteristics, their stratification, and relevant data.

To comprehensively examine potentially clinically significant characteristics, we analysed outcomes by considering all variables that could be derived from the included studies. Furthermore, we predefined three specific characteristics of interest:

1. Prior treatment with OAPs: Considering that both the pharmacokinetic and pharmacodynamic properties of a drug can impact the clinical response to an illness, and drawing insights from a prior study illustrating the influence of pre-treated OAPs in patients transitioning to risperidone LAI [26], we sought to investigate whether variations in OAP history might contribute to divergent clinical responses in patients undergoing a switch to PP1M.
2. Region: Previous studies have indicated that Asia has demonstrated a comparatively lower utilization rate of LAIs compared to other regions [16–18]. Therefore, our study sought to summarise evidence that investigated the efficacy of PP1M in Asia and specific Asian countries. The objective was to determine whether our findings could substantiate the clinical value and encourage the increased use of PP1M in the Asian context.
3. Stage of disease: Paliperidone palmitate injection has demonstrated efficacy in both the acute and maintenance phases of schizophrenia compared to a placebo in prior research [27, 28]. Many studies focusing on maintenance-phase enrolled patients with prior OAP treatment [28, 29]. Nevertheless, the extent of this benefit to patients in the acute phase, who have received prior treatment with OAPs, remains uncertain. Therefore, our objective was to systematically review existing evidence to ascertain whether the use of PP1M provides advantages to patients with a history of prior OAP treatment in both the acute and stable stages of the disease.

For PANSS, CGI-S and PSP scores, we presented their average changed data with standard deviation (mean difference ± SD) from a single study or the range of means extracted from several studies. We presented average endpoint data with their SD when changed data were not available (mean ± SD). For those that did not report average scores, we described the data as reported in the original studies, such as odds ratio (OR) or hazard ratio (HR) with their 95% confidence interval (95% CI) and *p* value. For dichotomous data (i.e., hospitalisation rate), we extracted and presented the reported proportion from original studies.

### Quality assessment

The quality of included studies was assessed independently by two reviewers. The Revised Cochrane risk-of-bias tool for randomised trials (RoB2) was employed to assess the risk of bias in RCTs [30]. The 9-point Newcastle-Ottawa Scale (NOS) instrument that contains 8 items was employed for non-randomised studies and cohort studies [31]. For before and after trials, we used the 12-item National Institutes of Health (NIH) quality assessment tool for before-after (Pre-Post) study without control group [32]. The risk of bias of prognostic studies were assessed using the Quality in Prognosis Studies (QUIPS) tool [33].

## Results

### Study selection results and characteristics and quality of included studies

**Fig. 1:**
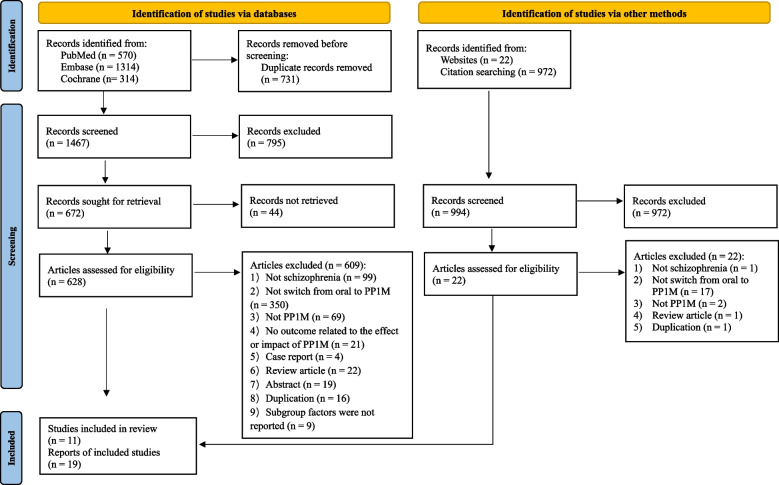
PRISMA study selection flowchart

An initial search retrieved 3192 records; removal of duplicates resulted in 2461 records for review. Of these, 1767 were considered ineligible and removed. The remaining 694 records were searched for full text, and 44 of them were not obtained due to unauthorised access. Among the 650 records that assessed for eligibility, 631 records were excluded with reasons illustrated in Fig. 1. Finally, a total of 11 studies with 19 references were included [6, 24, 29, 34–49]. The 11 included studies with 4150 patients comprise 5 pre-post trials [24, 38, 43, 45, 48], 4 observational trials [6, 40–42], 1 randomised controlled trial (RCT) [34], and 1 post hoc RCT [29]. The sample size ranged from 12 to 1166. Detailed characteristics and overall quality assessment result of included studies are shown in Table 1. For assessment details pertaining to RoB2, NOS, NIH, and QUIPS tool, see Supplementary 1.

**Table 1:** Characteristics of included studies

**Study**	**Country**	**Study design**	**Sample size**	**Outcomes**	**Other information**	**Quality assessment by RoB2**^**c**^	**Quality assessment by NOS**^**d**^	**Quality assessment by NIH**^**e**^	**Quality assessment by QUIPS**^**f**^
Bozzatello 2019 [34]	Italy	Randomized controlled trial	33	CGI-S, PSP	ACTRN12618001113246	High	NA	NA	NA
Kim 2021 [6]	Korea	Observational study	1,166	CGI-S, PSP	Post marketing surveillance data	NA	NA	NA	High
Li 2016 [37, 38, 47, 49]	Asia	Pre-post trial	212	PANSS, CGI-S, PSP	NCT01527305	NA	NA	Good	NA
Li 2018 [24]	China	Pre-post trial (Multivariate analysis)	610	PANSS, CGI-S, PSP	NCT01685931 post hoc analysis during acute treatment phase	NA	NA	NA	Low
Magliocco 2020 [40]	Italy	Observational study	12	PANSS, PSP	Real-word study	NA	8	NA	NA
Patel 2020 [41]	USA	Observational study	177	Hospitalization rate	Real-word analysis	NA	NA	Fair	NA
Peitl 2022 [42]	Croatia	Observational study	112	CGI-S	Real-word study	NA	NA	Fair	NA
Schreiner 2014 [35, 36, 43, 44]	21 countries^a^	Pre-post trial	593	PANSS, CGI-S, PSP	NCT01281527 subset of nonacute patients	NA	NA	Fair	NA
Si 2016 [45]	China	Pre-post trial	608	PANSS, CGI-S, PSP	NCT01685931 acute treatment phase	NA	NA	Fair	NA
Sliwa 2011 [29]	USA, Europe, and Asia	Post hoc RCT	106	PANSS, CGI-S, PSP	NCT00590577 post hoc analysis	Low	NA	NA	NA
Zhang 2015 [39, 46, 48, 49]	Asia–Pacific^b^	Pre-post trial	521	PANSS, CGI-S, PSP Hospitalization rate	NCT01051531	NA	NA	Fair	NA

*Abbreviations*: *CGI-S* Clinical global impression-severity, *NA* Not applicable, *NIH* National Institutes of Health, *NOS* Newcastle-Ottawa Scale, *PANSS* Positive and negative syndrome scale, *PSP* Personal and social performance, *RCT* Randomised controlled trial, *RoB2* Risk of bias tool 2

^a^21 countries included Austria, Belgium, Croatia, Denmark, Estonia, France, Germany, Greece, Hungary, Israel, Italy, Latvia, Lithuania, The Netherlands, Portugal, Spain, Sweden, Switzerland, Turkey, Ukraine, and the United Kingdom

^b^87.2% patients from Asian countries and 12.8% from Australia and New Zealand

^c^Quality assessment results of RCTs using RoB2. Overall quality is rated as low, high, or unclear. ‘NA’ in this column means the study is not an RCT and is not applicable to be assessed using RoB2

^d^Quality assessment results of non-randomised trials and cohort studies using NOS. The maximum score is 9. Higher score indicates better quality. ‘NA’ in this column means the study is not a cohort study and thus is not applicable to be assessed using NOS

^e^Quality assessment results of before and after trials using NIH. Quality was rated as poor (0-4 out of 12 questions), fair (5-8 out of 12 questions), or good (9-12 out of 12 questions). ‘NA’ in this column means the study is not a before and after study and thus is not applicable to be assessed using NIH

^f^Quality assessment results of prognostic studies using QUIPS. ‘NA’ in this column means the study is not a prognostic study and thus is not applicable to be assessed using QUIPS

### Patient characteristics that identified from included studies

Throughout the 11 included studies, we identified 9 characteristics as listed below:

1. prior treatment with OAPs, stratified into risperidone (RIS), olanzapine (OLA), aripiprazole (ARI), paliperidone, paliperidone extended-release (Pali ER), quetiapine (QUE), or other OAPs;
2. region, stratified into Asia-Pacific region, Asian, or Chinese patients;
3. stage of disease, stratified into acute or stable patients;
4. duration of illness (DI), stratified into ≤ 3 years, > 3 years, 3 < DI ≤10 years, > 10 years, ≤ 5 years or > 5 years;
5. reason for switching to PP1M, stratified into switching due to lack of efficacy or switching due to other reasons;
6. history of hospitalisation, with only one stratification was identified from included studies, which was equal to or greater than one time;
7. time of starting injection of PP1M, stratified into ≤ 1 week and > 1 week from date of hospitalisation;
8. PANSS total score at baseline, as a continuous stratification;
9. PSP total score at baseline, as a continuous stratification.

Notably, not all included studies reported the predefined outcomes, and not every outcome measure included all 9 characteristics and their stratification.

### PANSS score

A total of 7 studies 2662 patients (5 pre-post trials, 1 post hoc RCT and 1 observational study) reported the average PANSS score as an outcome measure (Table 2) [29, 35–38, 40, 44–48].

**Table 2:** Summary of characteristics identified from included studies reporting PANSS score

**Characteristics categories**	**Stratifications**	**Study ID**	**Study design**	**Sample size**	**PANSS score** (mean ± SD)
Prior treatment with OAP	Risperidone (RIS)	Schreiner 2014 [44]	Pre-post trial (NCT01281527)	191	Baseline: 70.8 ± 15.1; Endpoint: 56.9 ± 17.3; Change: -13.9 ± 14.8
		Si 2016 [45]	Pre-post trial (NCT01685931)	263	Baseline: 91.8 ± 12.4; Change: -31.0 ± 18.4
		Sliwa 2011 [29]	Post hoc RCT (NCT00590577)	106	Baseline: 87.95 ± 12.13; Change: -16.62 ± 22.33
	Olanzapine (OLA)	Schreiner 2014 [44]	Pre-post trial (NCT01281527)	87	Baseline: 71.4 ± 13.2; Endpoint: 62.3 ± 19.6; Change: -9.1 ± 17.5
		Si 2016 [45]	Pre-post trial (NCT01685931)	52	Baseline: 87.7 ± 12.5; Change: -25.5 ± 20.0
	Aripiprazole (ARI)	Schreiner 2014 [44]	Pre-post trial (NCT01281527)	46	Baseline: 74.7 ± 14.9; Endpoint: 62.6 ± 16.5; Change: -12.2.± 16.7
	Paliperidone extended-release (Pali ER)	Schreiner 2014 [44]	Pre-post trial (NCT01281527)	104	Baseline: 71.3 ± 14.3; Endpoint: 60.4 ± 17.2; Change: -10.8 ± 14.4
	Paliperidone	Magliocco 2020 [40]	Observational study	12	Baseline: 98.33 ± 15.01; Endpoint-Mean: 72.626
	Quetiapine (QUE)	Schreiner 2014 [44]	Pre-post trial (NCT01281527)	44	Baseline: 70.8 ± 13.1; Endpoint: 60.5 ± 20.1; Change: -10.2 ± 19.6
	Other (chlorpromazine, haloperidol, penfluridol, perphenazine, sulpiride, aripiprazole, ziprasidone, amisulpride, quetiapine fumarate, amisulpride, clozapine)	Si 2016 [45]	Pre-post trial (NCT01685931)	293	Baseline: 92.4 ± 12.5; Change: -31.7 ± 20.4
Ethnicity	Asia-Pacific region patients	Zhang 2015 [48]	Pre-post trial (NCT01051531)	521	Baseline: 64.1 ± 19.09; Change: -11.3 ± 21.38
	Asian patients	Li 2016 [38]	Pre-post trial (NCT01527305)	212	Baseline: 90.0 ± 17.41; Change: -23.9 ± 23.24
	Chinese Patients	Zhang 2015 [46]	Pre-post trial (NCT01051531)	108	Baseline: 67.6 ± 18.44; Change: -15.3 ± 20.76
		Si 2016 [45]	Pre-post trial (NCT01685931)	608	Baseline: 91.74 ± 12.43; Change: -30.87 ± 19.48
Stage of disease	Acute Patients	Schreiner 2014 [36]	Pre-post trial (NCT01281527)	212	Baseline: 98.5 ± 20.1; LOCF Endpoint: 67.4 ± 24.0; Change: -31.0 ± 29.0
		Li 2016 [38]	Pre-post trial (NCT01527305)	212	Baseline: 90.0 ± 17.41; Change: -23.9 ± 23.24
		Sliwa 2011 [29]	Post hoc RCT (NCT00590577)	106	Baseline: 87.95 ± 12.13; Change: -16.62 ± 22.33
		Si 2016 [45]	Pre-post trial (NCT01685931)	608	Baseline: 91.74 ± 12.43; Change: -30.87 ± 19.48
	Stable Patients	Schreiner 2014 [35]	Pre-post trial (NCT01281527)	589	Baseline: 71.45 ± 14.53; Endpoint: 59.75 ± 17.99; Change: -11.76 ± 15.63
		Schreiner 2014 [43]	Pre-post trial (NCT01281527)	593	Baseline: 71.5 ± 14.6; Change: -11.7 ± 15.9; LOCF Endpoint: 59.7 ± 18.1
Duration of illness	≤5 years	Li 2016 [47]	Pre-post trial (NCT01527305)	88	Baseline: 90.6 ± 14.04; Change: -30.0 ± 20.84
		Zhang 2015 [48]	Pre-post trial (NCT01051531)	521	Baseline: 64.1 ± 19.09; Change: -11.3 ± 21.38
	>5 years	Li 2016 [47]	Pre-post trial (NCT01527305)	124	Baseline: 89.5 ± 19.49; Change: -19.6 ± 20.99
	≤3 years	Schreiner 2014 [35]	Pre-post trial (NCT01281527)	231	Baseline: 72.6 ± 14.8; Endpoint: 57.5 ± 16.9; Change: -15.1 ± 15.6*
	>3 years	Schreiner 2014 [35]	Pre-post trial (NCT01281527)	358	Baseline: 70.7 ± 14.4; Endpoint: 61.2 ± 18.7; Change: -9.6 ± 15.7*
	>3 years vs ≤3 years	Li 2018 [24]	Pre-post trial (Multivariate analysis)	NR	PANSS<70: Odds Ratio (95%CI): 0.56 (0.34-0.92), *p*<0.0211*
Reason for switching PP1M	Switched for Lack of Efficacy	Schreiner 2014 [43]	Pre-post trial (NCT01281527)	144	Baseline: 80.3 ± 11.3; Change: -12.1 ± 15.1; LOCF Endpoint: 68.2 ± 17.0
	Switched for Other Reasons	Schreiner 2014 [43]	Pre-post trial (NCT01281527)	449	Baseline: 68.6 ± 14.4; Change: -11.6 ± 16.2; LOCF Endpoint: 57.0 ± 17.6
Time of start injection of PP1M	≤1 week	Li 2016 [37]	Observational study	121	Baseline: 89.1 ± 14.99; Change: -26.4 ± 19.38*
	>1 week	Li 2016 [37]	Observational study	91	Baseline: 91.1 ± 20.21; Change: -20.6 ± 27.31*
PANSS total score at baseline	Continuous measures	Li 2018 [24]	Pre-post trial (Multivariate analysis)	NR	PANSS<70: Odds Ratio (95%CI): 0.91 (0.88-0.93), *p*<0.0001*

*LOCF* Last observation carried forward, *NR* Not reported, *OAP* Oral antipsychotic, *PANSS* Positive and Negative Syndrome Scale, *PP1M* Once-monthly paliperidone palmitate, *RCT* Randomised controlled trial

^*^statistically significant difference between groups within the same study, *p*≤0.05

#### Prior treatment with OAPs

Of the 7 studies, 4 studies with 1319 patients (2 pre-post trials, 1 post hoc RCT, 1 observational study) provided information on patient’s prior treatment with OAPs [29, 40, 44, 45]. Among patients treated with RIS and OLA [29, 44, 45], the average change in PANSS score ranged between -31.0 and -13.9, and -25.5 and -9.1, respectively. The average changed PANSS score for patients with ARI, Pali ER, QUE, and other OAPs was -12.2 ± 16.7, -10.8 ± 14.4, -10.2 ± 19.6, and -31.7 ± 20.4, respectively [44, 45]. One study reported that the average endpoint PANSS score for patients previously treated with paliperidone was 72.626 [40].

#### Region

Three pre-post trials with 1341 patients reported the region of the patients [38, 45, 46, 48]. For Asia-Pacific patients, the average change in PANSS score was -11.3 ± 21.38 [48]. On the other hand, for Asian patients, the average change in PANSS score was -23.9 ± 23.24 [38]. The average change in PANSS score for Chinese patients ranged between -30.87 and -15.3 [45, 46].

#### Stage of disease

Moreover, 4 studies including three pre-post trials and one post hoc RCT with a total of 1519 patients reported stage of disease [29, 35, 36, 38, 43, 45]. The average change in PANSS score for acute patients ranged from -31.0 to -16.62 [29, 36, 38, 45]. On the other hand, for stable patients, the average change in PANSS score was -11.76 [35, 43].

#### Duration of illness

The duration of illness was reported in 4 pre-post trials with 1936 patients [24, 35, 47, 48]. For patients with a DI ≤ 5 years, the average change in PANSS score ranged between -30.0 and -11.3 [47, 48]. In patients with a DI > 5 years, the average change in PANSS score was -19.6 ± 20.99 [47]. One trial compared the change in PANSS score between patients with a DI ≤ 3 years and > 3 years [35]. Results demonstrated a significantly greater change in PANSS score in patients with a DI ≤ 3 years compared to those with a DI > 3 years (-15.1 ± 15.6 vs. -9.6 ± 15.7, *p* < 0.0001). Another trial also observed a significant difference in the change in PANSS score between patients with a DI > 3 years and those with a DI ≤ 3 years (OR 0.56, 95% CI 0.34 – 0.92, *p*<0.0211) [24].

#### Reasons for switching to PP1M

In a pre-post trial with 593 patients, the average change in PANSS score was -12.1 ± 15.1 for patients switched due to lack of efficacy [43]. For patients switched for other reasons, the average change in PANSS score was -11.6 ± 16.2 [43].

#### Time of starting injection of PP1M

According to an observational study involving 212 patients, the group that started the injection within ≤ 1 week (-26.4 ± 19.38) exhibited a significantly greater change in PANSS score compared to the group that started the injection after > 1 week (-20.6 ± 27.31) (between-group test: -6.9 ± 2.98, 95% CI -12.81 to -1.07, p ≤ 0.05) [37].

#### PANSS total score at baseline

A pre-post trial with 610 patients reported that PANSS total score at baseline was associated with less probability of endpoint PANSS score < 70 (OR 0.91, 95% CI 0.88 – 0.93, *p* < 0.0001) [24].

### CGI-S score

A total of 8 studies with 3351 patients (4 pre-post trials, 2 observational studies, 1 RCT and 1 post hoc RCT) reported the average CGI-S score as an outcome measure (Table 3) [6, 24, 29, 34, 36, 38, 42–48].

**Table 3:** Summary of characteristics identified from included studies reporting CGI-S score

**Subgroup factors**	**Stratification factor**	**Study ID**	**Study design**	**Sample size**	**CGI score** (mean ± SD)
Prior treatment with OAP	Risperidone (RIS)	Schreiner 2014 [44]	Pre-post trial (NCT01281527)	191	Baseline: 3.8 ± 0.9; Endpoint: 3.0 ± 1.0; Change: -0.8 ± 0.9
		Si 2016 [45]	Pre-post trial (NCT01685931)	263	Baseline: 5.2 ± 0.7; Change: -1.8 ± 1.3
		Sliwa 2011 [29]	Post hoc RCT (NCT00590577)	106	Baseline: 4.58 ± 0.69; Change: -0.99 ± 1.45
	Olanzapine (OLA)	Schreiner 2014 [44]	Pre-post trial (NCT01281527)	87	Baseline: 3.7 ± 1.0; Endpoint: 3.3 ± 1.2; Change: -0.4 ± 1.1
		Si 2016 [45]	Pre-post trial (NCT01685931)	52	Baseline: 5.3 ± 0.7; Change: -1.7 ± 1.3
	Aripiprazole (ARI)	Schreiner 2014 [44]	Pre-post trial (NCT01281527)	46	Baseline: 4.1 ± 0.8; Endpoint: 3.5 ± 1.0; Change: -0.6 ± 1.1
	Paliperidone extended-release (Pali ER)	Schreiner 2014 [44]	Pre-post trial (NCT01281527)	104	Baseline: 3.9 ± 0.9; Endpoint: 3.4 ± 1.1; Change: -0.6 ± 1.1
	Quetiapine (QUE)	Schreiner 2014 [44]	Pre-post trial (NCT01281527)	44	Baseline: 3.9 ± 0.9; Endpoint: 3.4 ± 1.0; Change: -0.5 ± 1.1
	Other (chlorpromazine, haloperidol, penfluridol, perphenazine, sulpiride, aripiprazole, ziprasidone, amisulpride, quetiapine fumarate, amisulpride, clozapine)	Si 2016 [45]	Pre-post trial (NCT01685931)	293	Baseline: 5.3 ± 0.7; Change: -1.9 ± 1.3
Ethnicity	Asia-Pacific region patients	Zhang 2015 [48]	Pre-post trial (NCT01051531)	521	Baseline: 3.4 ± 1.10; Change: -0.8 ± 1.35
	Asian patients	Li 2016 [38]	Pre-post trial (NCT01527305)	212	Baseline: 4.9 ± 0.79; Change: -1.4 ± 1.33
	Chinese Patients	Zhang 2015 [46]	Pre-post trial (NCT01051531)	108	Baseline: 3.8 ± 1.15; Change: -1.2 ± 1.54
		Si 2016 [45]	Pre-post trial (NCT01685931)	608	Baseline: 5.26 ± 0.70; Change: -1.84 ± 1.30
Stage of disease	Acute Patients	Peitl 2022 [42]	Observational study	112	Baseline: 5.2 ± 0.8; Endpoint: 2.6 ± 0.5
		Schreiner 2014 [36]	Pre-post trial (NCT01281527)	212	Baseline: 5.0 ± 0.8; LOCF Endpoint: 3.5 ± 1.3; Change: -1.5 ± 1.3
		Li 2016 [38]	Pre-post trial (NCT01527305)	212	Baseline: 4.9 ± 0.79; Change: -1.4 ± 1.33
		Si 2016 [45]	Pre-post trial (NCT01685931)	608	Baseline: 5.26 ± 0.70; Change: -1.84 ± 1.30
		Sliwa 2011 [29]	Post hoc RCT (NCT00590577)	106	Baseline: 4.58 ± 0.69; Change: -0.99 ± 1.45
	Stable Patients	Bozzatello 2018 [34]	RCT (ACTRN12618001113246)	33	Baseline: 4.90 ± 0.82; Endpoint: 4.16 ± 1.21
		Schreiner 2014 [44]	Pre-post trial (NCT01281527)	472	Baseline: 3.84 ± 0.90; Endpoint: 3.23 ± 1.05; Change: -0.63 ± 1.02
		Schreiner 2014 [43]	Pre-post trial (NCT01281527)	593	Baseline: 3.9 ± 0.9; Change: -0.6 ± 1.0; LOCF Endpoint: 3.3 ± 1.1
Duration of illness (DI)	DI ≤ 3 years	Kim 2021 [6]	Observational study	240	The change in CGI-S score was significantly different according to the DI and those with DI less than 3 years showed the most improvement in the aspect of clinical symptoms (DI, *p*<0.001; week, *p*<0.001; DI*week, *p*=0.013)*
	3 < DI ≤10 years	Kim 2021 [6]	Observational study	442	
	DI > 10 years	Kim 2021 [6]	Observational study	484	
	≤5 years	Li 2016 [47]	Pre-post trial (NCT01527305)	88	Baseline: 5 ± 0.70; Endpoint: 3.2 ± 1.15; Change: -1.8 ± 1.25*
		Zhang 2015 [48]	Pre-post trial (NCT01051531)	521	Baseline: 3.4 ± 1.10; Change: -0.8 ± 1.35
	>5 years	Li 2016 [47]	Pre-post trial (NCT01527305)	124	Baseline: 4.8 ± 0.85; Endpoint: 3.7 ± 1.32; Change: -1.1 ± 1.32*
Reason for switching PP1M	Switched for Lack of Efficacy	Schreiner 2014 [43]	Pre-post trial (NCT01281527)	144	Change: -0.6 ± 0.9
	Switched for Other Reasons	Schreiner 2014 [43]	Pre-post trial (NCT01281527)	449	Change: -0.6 ± 1.1
Time of start injection of PP1M	≤1 week	Li 2016 [37]	Observational study	121	Baseline: 4.9 ± 0.78; Change: -1.5 ± 1.21
	>1 week	Li 2016 [37]	Observational study	91	Baseline: 4.9 ± 0.82; Change: -1.3 ± 1.48

*LOCF* Last observation carried forward, *OAP *Oral antipsychotic, *CGI-S* Clinical Global Impressions – Severity, *PP1M* Once-monthly paliperidone palmitate, *RCT* Randomised controlled trial

^*^statistically significant difference between groups within the same study, *p*≤0.05

#### Prior treatment with OAPs

Three studies with 1307 patients (two pre-post trials and one post hoc RCT) reported the prior treatment with OAPs [29, 44, 45]. Among patients with RIS and OLA, the average change in CGI-S score ranged between -1.8 to -0.8 and -1.7 and -0.4, respectively [29, 44, 45]. For patients with ARI, Pali ER, QUE, and other OAPs, the average change in CGI-S score was -0.6 ± 1.1, -0.6 ± 1.1, -0.5 ± 1.1, and -1.9 ± 1.3, respectively [44, 45].

#### Region

Three pre-post trials with 1341 patients reported patient’s region [38, 45, 46, 48]. Among patients from the Asia-Pacific region, the average change in CGI-S score was -0.8 ± 1.35 [48]. For Asian patients, the average change was -1.4 ± 1.33, respectively [38]. In the case of Chinese patients, the average change ranged from -1.84 to -1.2 [45, 46].

#### Stage of disease

We identified 6 studies with a total of 1664 patients (three pre-post trials, one RCT, one post hoc RCT and one observational study) reported the stage of disease [29, 34, 36, 38, 42–45]. Among acute patients, four studies reported the average change in CGI-S score, which ranged between -1.84 and -0.99 [29, 36, 38, 45]. Instead of providing the changed score, one study presented the endpoint score, which was reported as 2.6 ± 0.5 [42]. For stable patients, one study reported the average change in CGI-S score was -0.63 ± 1.02 [43, 44]. The other study provided the endpoint score, reported as 4.16 ± 1.21 [34].

#### Duration of illness

Three studies with a total of 1899 patients (two pre-post trials and one observational study) reported DI [6, 47, 48]. In an observational study, it was reported that the change in CGI-S score for patients with DI ≤ 3 years was significantly greater than those with a DI of 3 < DI ≤10 years and DI > 10 years (*p* < 0.001) [6]. In a pre-post trial that compared the change in CGI-S score between patients with a DI ≤ 5 years and > 5 years, the results showed that patients with a DI ≤ 5 years demonstrated significantly better improvement compared to those with a DI > 5 years (*p* = 0.0008) [47]. Another pre-post trial reported the average change in CGI-S score for patients with a DI ≤ 5 years was -0.8 ± 1.35 [48].

#### Reasons for switching to PP1M

The reasons for switching to PP1M was provided in one pre-post trial with 593 patients [43]. The results showed that the average change in CGI-S score was similar for patients switching due to lack of efficacy or for other reasons (-0.6 ± 0.9 vs. -0.6 ± 1.1, *p* = 0.7621).

#### Time of starting injection of PP1M

One observational study with 212 patients provided information on the time of starting injection of PP1M [37]. The results demonstrated that the difference in average change in CGI-S score was similar between patients that started the injection within 1 week and after 1 week (-1.5 ± 1.21 vs. -1.3 ± 1.48).

### PSP score

A total of nine studies with 3861 patients (5 pre-post trials, 2 observational studies, 1 RCT and 1 post hoc RCT) reported the average PSP scores as an outcome measure (Table 4) [6, 24, 29, 34–38, 40, 43–47, 49].

**Table 4:** Summary of characteristics identified from included studies reporting PSP score

**Subgroup factors**	**Stratification factor**	**Study ID**	**Study design**	**Sample size**	**PSP score** (mean ± SD)
Prior treatment with OAP	Risperidone (RIS)	Schreiner 2014 [44]	Pre-post trial (NCT01281527)	191	Baseline: 57.8 ± 12.3; Endpoint: 68.2 ± 13.9; Change: 10.4 ± 13.8
		Si 2016 [45]	Pre-post trial (NCT01685931)	263	Baseline: 45.0 ± 13.6; Change: 19.5 ± 15.9
		Sliwa 2011 [29]	Post hoc RCT (NCT00590577)	106	Baseline: 50.76 ± 12.05; Change: 11.70 ± 16.96
	Olanzapine (OLA)	Schreiner 2014 [44]	Pre-post trial (NCT01281527)	87	Baseline: 61.5 ± 14.6; Endpoint: 66.0 ± 17.7; Change: 4.5 ± 15.9
		Si 2016 [45]	Pre-post trial (NCT01685931)	52	Baseline: 43.8 ± 14.2; Change: 17.1 ± 17.2
	Aripiprazole (ARI)	Schreiner 2014 [44]	Pre-post trial (NCT01281527)	46	Baseline: 58.9 ± 13.4; Endpoint: 62.9 ± 15.2; Change: 3.9 ± 13.2
	Paliperidone	Magliocco 2020 [40]	Observational study	12	Baseline: 46.75 ± 10.50; Endpoint-Mean: 59.75
	Paliperidone extended-release (Pali ER)	Schreiner 2014 [44]	Pre-post trial (NCT01281527)	104	Baseline: 58.3 ± 13.7; Endpoint: 65.4 ± 16.4; Change: 7.0 ± 13.8
	Quetiapine (QUE)	Schreiner 2014 [44]	Pre-post trial (NCT01281527)	44	Baseline: 56.3 ± 12.0; Endpoint: 64.2 ± 15.9; Change: 7.9 ± 12.4
	Other (chlorpromazine, haloperidol, penfluridol, perphenazine, sulpiride, aripiprazole, ziprasidone, amisulpride, quetiapine fumarate, amisulpride, clozapine)	Si 2016 [45]	Pre-post trial (NCT01685931)	293	Baseline: 44.9 ± 13.6; Change: 19.6 ± 16.5
Ethnicity	Asia-Pacific region patients	Zhang 2015 [49]	Pre-post trial (NCT01051531)	516	Baseline: 58.5 ± 16.18; Change: 10.5 ± 19.55
	Asian patients	Li 2016 [37]	Observational study	212	Baseline: 42.81 ± 13.07; Change: 18.86 ± 17.48
		Li 2016 [49]	Pre-post trial (NCT01527305)	212	Baseline: 42.83 ± 13.11; Change: 18.88 ± 16.66
		Li 2016 [38]	Pre-post trial (NCT01527305)	212	Baseline: 42.8 ± 13.14; Change: 18.8 ± 17.56
	Chinese Patients	Zhang 2015 [46]	Pre-post trial (NCT01051531)	108	Baseline: 53.8 ± 16.03; Change: 15.9 ± 19.65
		Si 2016 [45]	Pre-post trial (NCT01685931)	608	Baseline: 44.85 ± 13.62; Change: 19.34 ± 16.26
Stage of disease	Acute Patients	Li 2016 [37]	Observational study	212	Baseline: 42.81 ± 13.07; Change: 18.86 ± 17.48
		Li 2016 [49]	Pre-post trial (NCT01527305)	212	Baseline: 42.83 ± 13.11; Change: 18.88 ± 16.66
		Li 2016 [38]	Observational study	212	Baseline: 42.8 ± 13.14; Change: 18.8 ± 17.56
		Schreiner 2014 [36]	Pre-post trial (NCT01281527)	212	Baseline: 43.9 ± 15.0; LOCF Endpoint: 62.9 ± 17.1; Change: 19.0 ± 18.7
		Si 2016 [45]	Pre-post trial (NCT01685931)	608	Baseline: 44.85 ± 13.62; Change: 19.34 ± 16.26
		Sliwa 2011 [29]	Post hoc RCT (NCT00590577)	106	Baseline: 50.76 ± 12.05; Change: 11.70 ± 16.96
	Stable Patients	Bozzatello 2018 [34]	RCT (ACTRN12618001113246)	33	Baseline: 52.81 ± 6.82; Endpoint: 65.22 ± 9.64
		Schreiner 2014 [44]	Pre-post trial (NCT01281527)	472	Baseline: 58.56 ± 13.06; Endpoint: 66.28 ± 15.43; Change: 7.70 ± 13.94
		Schreiner 2014 [35]	Pre-post trial (NCT01281527)	589	Baseline-Mean: 58.11; Endpoint-Mean: 66.06
		Schreiner 2014 [43]	Pre-post trial (NCT01281527)	593	Baseline: 58.1 ± 13.4; LOCF Endpoint: 66.1 ± 15.7
Duration of illness (DI)	DI ≤ 3 years	Kim 2021 [6]	Observational study	240	All three groups showed significant improvements in PSP scores after the treatment with Paliperidone LAI and patients with DI less than 3 years demonstrated the highest PSP scores (DI, *p*<0.001; DI*week, *p*=0.436; week, *p*<0.001) *.
	3 < DI ≤10 years	Kim 2021 [6]	Observational study	442	
	DI > 10 years	Kim 2021 [6]	Observational study	484	
	≤5 years	Li 2016 [47]	Pre-post trial (NCT01527305)	88	Baseline: 42.6 ± 13.13; Change: 25.4 ± 16.22*
		Zhang 2015 [49]	Pre-post trial (NCT01051531)	516	Baseline: 58.5 ± 16.18; Change: 10.5 ± 19.55
	>5 years	Li 2016 [47]	Pre-post trial (NCT01527305)	124	Baseline: 43.0 ± 13.21; Change: 14.4 ± 17.11*
	≤3 years	Schreiner 2014 [35]	Pre-post trial (NCT01281527)	231	Baseline-Mean: 59.2; Endpoint-Mean: 67.7
	>3 years	Schreiner 2014 [35]	Pre-post trial (NCT01281527)	358	Baseline-Mean: 57.4; Endpoint-Mean: 65.0
Reason for switching PP1M	Switched for Lack of Efficacy	Schreiner 2014 [43]	Pre-post trial (NCT01281527)	144	Change: 5.5 ± 12.3*;LOCF Baseline: 55.3 ± 12.3
	Switched for Other Reasons	Schreiner 2014 [43]	Pre-post trial (NCT01281527)	449	Change: 8.8 ± 14.4*;LOCF Baseline: 59.0 ± 13.6
Time of start injection of PP1M	≤1 week	Li 2016 [37]	Observational study	121	Baseline: 43.8 ± 12.27; Change: 19.8 ± 16.50*
	>1 week	Li 2016 [37]	Observational study	91	Baseline: 41.5 ± 14.19; Change: 17.6 ± 18.89*
History of hospitalization	≥1	Li 2016 [37]	Observational study	212	Baseline: 42.81 ± 13.07; Change: 18.86 ± 17.48
PANSS total score at baseline	Continuous measures	Li 2018 [24]	Pre-post trial (Multivariate analysis)	NR	PSP>70: Odds Ratio (95%CI): 0.97 (0.96-0.99), *p*= 0.0102*
PSP total score at baseline	Continuous measures	Li 2018 [24]	Pre-post trial (Multivariate analysis)	NR	PSP>70: Odds Ratio (95%CI): 1.07 (1.05-1.10), *p*<0.0001*

*LOCF* Last observation carried forward, *NR* Not reported, *OAP* Oral antipsychotic, *PSP* Personal and Social Performance, *PP1M* Once-monthly paliperidone palmitate, *RCT* Randomised controlled trial

^*^statistically significant difference between groups within the same study, *p*≤0.05

#### Prior treatment with OAPs

There were 4 studies with 1319 patients (three pre-post trials and one observational study) provided information on prior OAP treatments [29, 40, 44, 45]. Patients reported a baseline average PSP score varied between 43.8 and 61.5. For patients with RIS and OLA, the average change in PSP score ranged from 10.4 to 19.5, and from 4.5 to 17.1, respectively [29, 44, 45]. The average change for patients with ARI, Pali ER, QUE, and other OAPs was 3.9 ± 13.2, 7.0 ± 13.8, 7.9 ± 12.4, and 19.6 ± 16.5, respectively [44, 45]. The average endpoint PSP score for patients with paliperidone was 59.75 [40].

#### Region

We identified 3 studies with a total of 1341 patients (two pre-post trials and one observational study) reported information on region [37, 38, 45, 46, 49]. The average change in PSP score for Asia-Pacific region patients was 10.5 ± 19.55 [49]. For Asian and Chinese patients, the range of average change in PSP score was 18.8 to 18.88 [37, 38, 49], and 15.9 to 19.34 [45, 48], respectively.

#### Stage of disease

Five studies with 1552 patients (3 pre-post trials, 1 observational study and 1 RCT) provided information on stage of disease [29, 34–38, 43–45, 49]. For acute patients, the average change in PSP score ranged from 11.70 to 19.34 [29, 36–38, 45, 49]. For stable patients, the average endpoint PSP score ranged between 65.22 and 66.28 [34, 35, 43, 44].

#### Duration of illness

Four studies with 2492 patients (three pre-post trials and one observational study) provided information on patient’s DI [6, 35, 47, 49]. One observational study reported significant improvements in PSP scores for patients with a DI ≤ 3 years, 3 < DI ≤10 years, and > 10 years [6]. It also demonstrated that patients with a DI ≤ 3 years provided the highest PSP scores compared to those with a 3 < DI ≤10 years or > 10 years (*p* < 0.001). However, another pre-post trial observed no significant between-group change from baseline in PSP score between patients with a DI ≤ 3 years and > 3 years (average endpoint: 67.7 vs. 65.0, *p* = 0.27) [35]. In addition, a pre-post trial reported a significantly greater improvements in changed PSP score for patients with a DI ≤5 years (average change: 25.4 ± 16.22) compared to those with a DI >5 years (average change: 14.4 ± 17.11) (mean difference 10.4, 95% CI 6.14 – 14.73, *p* < 0.0001) [47]. Another pre-post trial reported that the average change in PSP score for patients with a DI ≤5 years was 10.5 ± 19.55 [49].

#### Reasons for switching PP1M

Information regarding the reasons for switching to PP1M was provided in one pre-post trial with 593 patients [43]. Results showed that the average change in PSP score was significantly higher in patients switched for other reasons (8.8 ± 14.4) compared with patients switched for efficacy reasons (5.5 ± 12.3, *p* < 0.05).

#### Time of starting injection of PP1M

An observational study with 212 patients reported the time of starting injection of PP1M [37]. Results showed that patients started injection within 1 week reported a significant greater average change in PSP scores compared with those started injection after 1 week (change score: 19.8 ± 16.50 vs. 17.6 ± 18.89, *p* ≤ 0.05).

#### History of hospitalisation

An observational study with 212 patients reported that the change in PSP score for patients with more than 1 hospitalisation was 18.86 ± 17.48 [37].

#### PANSS total score at baseline

A pre-post trial with 610 patients reported a lower PANSS total score at baseline was associated with better improvements in PSP scores (OR 0.97, 95% CI 0.96 – 0.99, *p* < 0.0102) [24].

#### PSP total score at baseline

Moreover, the pre-post trial with 610 patients reported a higher PSP total score at baseline was associated with better improvements in PSP score (OR 1.07, 95% CI 1.05 – 1.10, *p* < 0.0001) [24].

### Hospitalisation rate

Two studies with 698 patients (1 observational study and 1 pre-post trial) reported hospitalisation rate [39, 41, 46, 48].

#### History of hospitalisation

The observational study with 177 patients provided information on patients’ history of hospitalisation [41]. Results showed that for patients with ≥ 1 hospitalisation, the hospitalisation rate was 32.8%, which was 58 out of 177 patients.

#### Region

The pre-post trial with 521 patients provided information on region [39, 46, 48]. Results showed that among the three categories, Asian patients reported the lowest hospitalisation rate (i.e., 8%, 36 out of 470 patients) [39], followed by Chinese patients (i.e., 6.5%, 7 out of 108 patients) [46] and Asia-Pacific region patients (i.e., 8.8%, 46 out of 521 patients) [48].

#### Duration of illness

In addition, the pre-post trial with 521 patients reported that patients with a DI ≤ 5 years showed a hospitalisation rate of 8.8% (46 patients) that was reduced from a baseline rate of 35.9% (187 patients) [48].

No studies provided information on prior treatment with OAPs, and stage of disease.

## Discussion

### Summary of findings

This review summarised the characteristics of schizophrenia patients switching from OAPs to PP1M in 11 studies with 4150 patients. A total of 9 characteristics were identified throughout the included studies. The most commonly reported characteristics was patient’s prior treatment with OAPs, followed by the stage of disease and duration of illness (DI). Other identified characteristics included regions, the reason for switching to PP1M, history of hospitalisation, time of start injection of PP1M, the PANSS and PSP total score at baseline. The influence of these characteristics was summarised according to predefined outcomes.

Our results indicate that patients in the acute stage or those with a shorter illness duration may exhibit a tendency for more pronounced improvements in symptom reduction and disease severity. Patients who initiate PP1M injection early (i.e., within ≤ 1 week) may show a tendency toward enhanced improvements in symptoms and psychosocial function. Additionally, transitioning for reasons other than efficacy-related issues may also demonstrate a trend toward improved psychosocial function.

### Stage of disease

Our systematic review revealed an intriguing finding regarding the influence of disease stage on PANSS and CGI-S score reduction in patients transitioning from OAPs to PP1M treatment. Specifically, we observed that the acute stage of the disease might be associated with a more substantial reduction in PANSS and CGI-S scores compared to patients with stable stage of the disease. According to the minimum clinically important differences (MCID, necessitating a 15-point or greater improvement on PANSS score [50] or a 1-point or more improvement on CGI-S score from baseline [51]), patients in acute stage (i.e., change in PANSS score ranged between -16.62 to -31, change in CGI ranged between -0.99 and -1.5) seemed to report better clinical outcomes compared to those in the stable stage (i.e., change in PANSS -11.76, change in CGI-S -0.63). It is plausible that patients in the acute stage exhibit a higher level of treatment responsiveness, thereby showing a greater response to PP1M treatment. This finding may have significant clinical implications, as patients in the acute stage often experience more severe symptoms, and achieving symptom improvement is a primary treatment goal [52]. The transition to PP1M treatment during this stage appears to offer potential benefits in terms of symptom management, patient well-being and severity reduction. In addition, this finding may contribute to the expanding body of evidence supporting the use of PP1M therapy during the acute phase as recent evidence and guidelines suggest that LAIs must also be considered earlier in therapy [53, 54]. Despite the limited evidence and heterogeneity across the included studies, these findings somehow highlight the importance of considering disease stage when making treatment decisions and support the notion that initiating the transition to PP1M treatment during the acute stage may optimize symptom and severity reduction and improve patient outcomes.

### Duration of illness

Another notable characteristic identified in our systematic review was that patients with a DI of ≤3 years exhibited a more favourable response on reducing symptoms and disease severity when switching from OAPs to PP1M treatment. Results also indicated that a DI ≤5 years was associated with more reduction on CGI-S score compared to a DI of > 5 years. This finding may provide important implications for early intervention and treatment strategies in schizophrenia. Patients within the early stages of illness may be more responsive to interventions, and the transition to PP1M treatment during this critical period could lead to improved outcomes. This might be consistent with previous research on untreated psychosis where the shorter duration was associated with greater response to antipsychotic treatment [55]. It could be hypothesized that switching patients with a shorter duration of illness may have a higher likelihood of treatment responsiveness as well, potentially due to a less chronic and more reversible disease trajectory. Previous research showed that DI influences treatment response, suicidal risk and loss of social functioning in schizophrenia [56]. The finding in this review may add to this body that extrapolate the influence of DI to patients switching from OAPs to PP1M. It is worth noting, although the beneficial influence of a DI of ≤ 5 years was observed in PSP scores compared with a DI of > 5 years, another two studies that compared a DI of ≤ 3 years and > 3 years provided inconsistent results. Therefore, the beneficial influence of early interventions remains unclear on improving functioning. Nevertheless, our findings suggest that identifying patients with a shorter duration of illness, such as ≤3 years, and considering the transition to PP1M treatment may offer benefits in terms of symptom management and overall treatment outcomes.

### Prior treatment with OAPs

Our findings indicated the most commonly reported OAP was RIS, followed by OLA and other OAPs. Regarding the PANSS score, the improvements on patients with ARI, Pali ER, and QUE, which ranged from -10.2 to -12.2 with a baseline score ranged between 70.8 and 74.7, did not meet the criteria of better clinical outcomes (i.e., ≥ 15 points improvements [50]). Nevertheless, patients previously treated with RIS and OLA displayed a diverse range of enhancements in PANSS (RIS: -13.9 to -31.0, OLA: -9.1 to -25.5) and CGI-S scores (RIS: -0.8 to -1.8, OLA: -0.4 to -1.7). This variability may suggest that a subset of patients who had received prior treatment with RIS and OLA may experience improved clinical outcomes upon transitioning to PP1M. The improvements on CGI-S score among patients with ARI, Pali ER, and QUE were very similar, i.e., ranging from -0.5 to -0.6, which was less than published criteria of better outcomes (i.e., ≥1 point improvement from baseline [51]). The extensive variability in reported outcome improvements presents a challenge in offering a conclusive recommendation regarding whether prior treatment with these OAPs should be considered a beneficial factor in ameliorating symptoms or reducing severity. Noteworthy, some uncontrolled factors may introduce confounding influences when interpreting our findings. For example, baseline scores could introduce a potential bias on the outcomes, wherein a greater baseline score might be associated with a more substantial improvement. This has been directly supported by one of the included studies that PANSS score at baseline showed significant influence on treatment outcomes (OR 0.91, 95% CI 0.88 – 0.93, *p* < 0.0001) [24]. Additionally, there may be interplay and cross-effects among different patient characteristics, emphasizing the complexity of the relationships within the dataset.

### Reasons for switching PP1M

In addition, our finding observed that patients switched for other reasons may provide a higher score on PSP scale compared to patients switched for efficacy reasons (*p* < 0.01). However, the change in average PANSS score and CGI-S score was similar for patients switching due to lack of efficacy or for other reasons. Scores were similar no matter the reasons for switching PP1M on PANSS and CGI-S scores. Notably, this study included non-acute but symptomatic patients who were considered stable by clinicians. This may provide clinical implications on making treatment decisions that in the context of stable patients receiving OAP treatments, it may be advantageous to consider an earlier switch to PP1M before the occurrence of lack of efficacy, in order to facilitate an enhancement in psychosocial functioning. 

### Time of start injection of PP1M

Compared with treatment outcomes when the first injection of PP1M is more than a week after admission, our findings indicated patients whose first injection was less than 1 week after admission showed a greater improvement on symptoms that measured by PANSS total score and psychosocial function that measured by PSP total score. The improvements met the criteria of better clinical outcomes [50]. This is consistent with previous research where significant improvements of PP1M in psychotic symptoms were observed on day 8 without OAPs augmentation compared to OAPs and placebo [27, 57]. Previous pharmacokinetic studies may offer a pertinent explanation for the above finding where PP1M achieves therapeutic, steady—state plasma levels rapidly on initiation without the necessity of oral supplementation [58]. Thus, the finding may provide clinical implications for the treatment strategy that switching to PP1M may offer early symptomatic improvements in early initiation.

### Regions

Despite the relatively low utilisation rate of LAIs in Asia [18], our findings indicate that patients who switched from OAPs to PP1M reported improvements on severity, mental state and functional well-being. These improvements appear to reach the clinically significant threshold [50, 51]. In light of recognized challenges and misconceptions from both clinician and patient viewpoints regarding LAIs’ use, our results might imply a sustained enhancement in patients' attitudes, clinicians' knowledge and experience, as well as policy makers' and healthcare service providers' perspectives towards LAIs in Asia.

### Strengths and limitations

To the utmost extent of our current understanding, this review represents the initial endeavour to succinctly synthesise available evidence pertaining to the attributes of individuals diagnosed with schizophrenia who transition from OAPs to PP1M. Employing a methodical process encompassing systematic searching, rigorous selection, and meticulous evaluation of pertinent studies, our systematic reviews have furnished an all-encompassing and impartial overview of this subject matter. Furthermore, the scope of our review extends beyond prognostic investigations to encompass interventional studies, thus affording valuable insights. This comprehensive approach ensures an extensive comprehension of the patient characteristics potentially associated with the transition to PP1M.

Nevertheless, it is crucial to exercise caution when interpreting our findings and avoid overgeneralization, primarily due to the limited availability of evidence, the absence of consensus regarding superior or successful clinical outcomes, and the substantial heterogeneity observed among the included studies. Following an exhaustive systematic search and rigorous screening process, only a total of 12 studies were deemed eligible for inclusion, providing pertinent information on patient characteristics and reporting predefined outcomes. Among these studies, a mere 25% were originally designed to investigate influential prognostic factors [6, 24], while the remaining 75% constituted interventional studies focused on evaluating the effectiveness of interventions, specifically the transition from OAPs to PP1M. The scarcity of evidence significantly impedes our ability to conduct a precise analysis regarding the potential impact of each identified characteristic and their interactive influence on treatment outcomes. Additionally, a limited number of characteristics were identified that potentially hold sway over the frequency of hospitalizations.

Moreover, the considerable heterogeneity observed among the included studies has posed challenges in combining data pertaining to the same characteristic, and the limited number of available studies has hindered the possibility of conducting meta-analysis or more refined stratification. The inclusion of various study designs may also introduce heterogeneity in our analysis. While this diversity enriches the breadth of evidence, it also necessitates consideration of potential influences on result interpretation and generalisability. Variations in participant characteristics, outcome measures, and temporal factors across study designs may affect the generalizability of the conclusions to broader populations or clinical settings. As a result, rather than the casual relationships, this study was only able to provide indicative trends regarding the potential influence of specific characteristics on treatment outcomes.

Furthermore, owing to the restricted number of studies that specifically examined this particular topic and the absence of a consensus definition for a successful transition from OAPs to PP1M, this review relied on the criteria established in previous research during the discussion. However, given the significant heterogeneity observed among the studies, for example, a wide range of PANSS baseline scores spanning from 70.8 to 98.33 was reported across studies providing relevant information on prior treatment with OAPs on PANSS total score, it is imperative to further develop and refine the definition of a successful transition based on various outcome measures.

Therefore, it is vital that future research endeavours focus on conducting additional studies specifically aimed at examining the patient characteristics that have a positive impact on treatment outcomes or can serve as predictors of a successful transition from OAPs to PP1M in individuals with schizophrenia. Moreover, reaching a consensus and refining the definition of a successful transition from OAPs to PP1M, taking into account different outcome indicators, is essential to enable future studies to offer more precise and practical clinical recommendations regarding treatment strategies

## Conclusion

Our review identified nine potential patient characteristics that may have influence on treatment outcomes in patients with schizophrenia switching from OAPs to PP1M. The findings suggested that patients in acute stage or with a shorter duration of illness may have a trend to provide better improvements on reducing symptoms and disease severity. Patients with an early initiation of PP1M injection (i.e., < 1 week from date of hospital admission) may have a trend on improving symptoms and psychosocial function. Switching due to reasons other than lack of efficacy may have a trend on improving psychosocial function. The influence of other potential characteristics remains ambiguous and inconclusive. Subsequent investigations are warranted to corroborate these findings through studies exploring influencing factors.

### Supplementary Information

**Additional file 1: Table S1.** Quality assessment results of two RCTs using ROB2 tool. **Table S2.** Quality assessment results of one cohort study using NOS. **Table S3.** Quality assessment results of six before and after studies using NIH scale. **Table S4.** Quality assessment results of two prognostic studies using QUIPS tool.

## Data Availability

All data generated or analysed during this study are included in this published article.

## Abbreviations

ARI: Aripiprazole
CGI-S: Clinical Global Impressions – Severity
CCT: Controlled clinical trial
DI: Duration of illness
LAIs: Long-acting injectable antipsychotics
MeSH: Medical subject headings
OLA: Olanzapine
OAPs: Oral antipsychotics
Pali ER: Paliperidone extended-release
PP1M: Paliperidone palmitate
PSP: Personal and Social Performance
PANSS: Positive and Negative Syndrome Scale
PRISMA: Preferred reporting items for systematic review and meta-analyses
QUE: Quetiapine
RCT: Randomised controlled trial
RIS: Risperidone
