# Supplementary material for: Characteristics of patients with schizophrenia switching from oral antipsychotics to once-monthly paliperidone palmitate (PP1M): a systematic review
Source: BMC Psychiatry. 2024 Jan 19;24:57. doi: 10.1186/s12888-024-05508-6 (PMC10799459; doi:10.1186/s12888-024-05508-6)
Supplement: Supplementary file 1 — Additional file 1: Table S1. Quality assessment results of two RCTs using ROB2 tool. Table S2. Quality assessment results of one cohort study using NOS. Table S3. Quality assessment results of six before and after studies using NIH scale. Table S4. Quality assessment results of two prognostic studies using QUIPS tool. [file 12888_2024_5508_MOESM1_ESM.docx]

**Supplementary 1 Quality assessment results**

Table S1 Quality assessment results of two RCTs using ROB2 tool

| Study ID | Randomization process | Deviations from intended interventions | Missing outcome data | Measurement of the outcome | Selection of the reported result | Overall Bias |
| --- | --- | --- | --- | --- | --- | --- |
| Bozzatello 2019 | Low | Low | Low | High^d^ | Low | High |
| Sliwa 2011 | Low^a^ | Low^b^ | Low^c^ | Low^e^ | Low^f^ | Low |
| ^a^ Random sequences generated by computer-generated random numbers; reference to a random number table. Allocated by the coordinating center. And no imbalances between intervention and control group. ^b^ Double-blind RCT and all analyses were performed on the intent-to-treat (ITT) analysis set. Or open-label RCT but all patients were adherenced to intervention. ^c^ The outcome data was available for ITT population. ^d^ The method of measuring the outcome is appropriate.The measurement or ascertainment of the outcome have no differed between intervention groups. But the author did not describe the information of assessment of the outcome have been influenced by knowledge of intervention received. ^e^ The method of measuring the outcome is appropriate. The measurement or ascertainment of the outcome have no differed between intervention groups. The outcome assessors not aware of the intervention received by study participants. ^f^ Data analysis was performed according to the plan and all measured outcomes were reported.  *RCTs* randomised controlled trials, *ROB* risk of bias | | | | | | |

Table S2 Quality assessment results of one cohort study using NOS

| Study ID | Selection | | | | Comparability | Outcome | | | Overall score |
| --- | --- | --- | --- | --- | --- | --- | --- | --- | --- |
|  | Representativeness of the exposed cohort | Selection of the non exposed cohort | Ascertainment of exposure | Demonstration that outcome of interest was not present at start of study | Comparability of cohorts on the basis of the design or analysis | Assessment of outcome | Was follow-up long enough for outcomes to occur | Adequacy of follow up of cohorts |  |
| Magliocco 2020 | 0 | 1 | 1 | 1 | 2 | 1 | 1 | 1 | 8 |

*NOS* Newcastle-Ottawa Scale

Table S3 Quality assessment results of six before and after studies using NIH scale

| Study ID | Question/ objective | Participants | | | | | Outcomes | | | Statistical analysis | | | Overall |
| --- | --- | --- | --- | --- | --- | --- | --- | --- | --- | --- | --- | --- | --- |
|  | Was the study question or objective clearly stated? | Were eligibility/selection criteria for the study population prespecified and clearly described? | Were the participants in the study representative of those who would be eligible for the test/service/intervention in the general or clinical population of interest? | Were all eligible participants that met the prespecified entry criteria enrolled? | Was the sample size sufficiently large to provide confidence in the findings? | Was the test/service/intervention clearly described and delivered consistently across the study population? | Were the outcome measures prespecified, clearly defined, valid, reliable, and assessed consistently across all study participants? | Were the people assessing the outcomes blinded to the participants' exposures/interventions? | Was the loss to follow-up after baseline 20% or less? Were those lost to follow-up accounted for in the analysis? | Did the statistical methods examine changes in outcome measures from before to after the intervention? Were statistical tests done that provided p values for the pre-to-post changes? | Were outcome measures of interest taken multiple times before the intervention and multiple times after the intervention (i.e., did they use an interrupted time-series design)? | If the intervention was conducted at a group level (e.g., a whole hospital, a community, etc.) did the statistical analysis take into account the use of individual-level data to determine effects at the group level? |  |
| Li 2016 | Yes | Yes | No | Yes | Yes | Yes | Yes | No | Yes | Yes | Yes | NA | Good |
| Patel 2020 | Yes | Yes | Yes | Yes | NR | NR | Yes | NA | Yes | Yes | No | NA | Fair |
| Peitl 2022 | Yes | Yes | No | Yes | NR | Yes | Yes | NR | Yes | Yes | No | NA | Fair |
| Schreiner 2014 | Yes | Yes | No | Yes | Yes | Yes | Yes | No | Yes | Yes | No | NA | Fair |
| Si 2016 | Yes | Yes | No | No | NR | Yes | Yes | No | Yes | Yes | Yes | NA | Fair |
| Zhang 2015 | Yes | Yes | Yes | Yes | Yes | Yes | Yes | No | Yes | Yes | Yes | NA | Good |
| *NA* not applicable, *NIH* National Institutes of Health, *NR* not reported  Quality was rated as poor (0-4 out of 12 questions), fair (5-8 out of 12 questions), or good (9-12 out of 12 questions) | | | | | | | | | | | | | |

Table S4 Quality assessment results of two prognostic studies using QUIPS tool

| Study ID | Overall risk of bias | Study participants | Study attrition | Prognostic factor measurement | Outcome assessment | Study confounding | Statistical analysis and reporting |
| --- | --- | --- | --- | --- | --- | --- | --- |
| Li 2018 | High^a^ | Low^b^ | Low^c^ | High^d^ | Low^e^ | High^g^ | Low^h^ |
| Kim 2021 | High^a^ | Low^b^ | Low^c^ | High^d^ | High^f^ | High^g^ | High^i^ |
| ^a^ High risk, all or only 1 of the signalling questions under this heading answered "no".  ^b^ The samples are representative: the time and place of sample recruitment start and end are clearly defined, and the selection criteria of samples and controls are fully described. The baseline is well described.  ^c^ The loss rate was less than 20% or none.  ^d^ No definition of risk factors was found. The measurement method is unknown and the cut off value is not told.  ^e^ Outcome indicators are clearly defined. The measurement method is sufficiently effective and reliable. The measurement method is consistent.  ^f^ Outcome indicator definition and measurement method are not described.  ^g^ Confounding factors are not described.  ^h^ The method of multi-factor analysis is described. There is no selective reporting of results.  ^i^ Only the rates of the two groups were compared, and no multi-factor analysis was performed. There is no selective reporting of results. | | | | | | | |
